# The impact of the carbohydrate-binding module on how a lytic polysaccharide monooxygenase modifies cellulose fibers

**DOI:** 10.1186/s13068-024-02564-8

**Published:** 2024-08-24

**Authors:** Fredrik G. Støpamo, Irina Sulaeva, David Budischowsky, Jenni Rahikainen, Kaisa Marjamaa, Kristiina Kruus, Antje Potthast, Vincent G. H. Eijsink, Anikó Várnai

**Affiliations:** 1 Norwegian University of Life Sciences (NMBU), Ås, Norway; 2 University of Natural Resources and Life Sciences (BOKU), Vienna, Austria; 3 VTT Technical Research Centre of Finland, Espoo, Finland; 4 Aalto University, Espoo, Finland

**Keywords:** Cellulose, Enzymatic fiber engineering, Oxidation, AA9 LPMOs, Functional variation, CBM, Size exclusion chromatography, Carbonyl detection, Fluorescence

## Abstract

**Background:**

In recent years, lytic polysaccharide monooxygenases (LPMOs) that oxidatively cleave cellulose have gained increasing attention in cellulose fiber modification. LPMOs are relatively small copper-dependent redox enzymes that occur as single domain proteins but may also contain an appended carbohydrate-binding module (CBM). Previous studies have indicated that the CBM “immobilizes” the LPMO on the substrate and thus leads to more localized oxidation of the fiber surface. Still, our understanding of how LPMOs and their CBMs modify cellulose fibers remains limited.

**Results:**

Here, we studied the impact of the CBM on the fiber-modifying properties of *Nc*AA9C, a two-domain family AA9 LPMO from *Neurospora crassa*, using both biochemical methods as well as newly developed multistep fiber dissolution methods that allow mapping LPMO action across the fiber, from the fiber surface to the fiber core. The presence of the CBM in *Nc*AA9C improved binding towards amorphous (PASC), natural (Cell I), and alkali-treated (Cell II) cellulose, and the CBM was essential for significant binding of the non-reduced LPMO to Cell I and Cell II. Substrate binding of the catalytic domain was promoted by reduction, allowing the truncated CBM-free *Nc*AA9C to degrade Cell I and Cell II, albeit less efficiently and with more autocatalytic enzyme degradation compared to the full-length enzyme. The sequential dissolution analyses showed that cuts by the CBM-free enzyme are more evenly spread through the fiber compared to the CBM-containing full-length enzyme and showed that the truncated enzyme can penetrate deeper into the fiber, thus giving relatively more oxidation and cleavage in the fiber core.

**Conclusions:**

These results demonstrate the capability of LPMOs to modify cellulose fibers from surface to core and reveal how variation in enzyme modularity can be used to generate varying cellulose-based materials. While the implications of these findings for LPMO-based cellulose fiber engineering remain to be explored, it is clear that the presence of a CBM is an important determinant of the three-dimensional distribution of oxidation sites in the fiber.

**Supplementary Information:**

The online version contains supplementary material available at 10.1186/s13068-024-02564-8.

## Background

In 2010, oxidative cleavage of polysaccharides by enzymes that today are known as lytic polysaccharide monooxygenases (LPMOs) was first described by Vaaje-Kolstad et al. [1]. One of the first LPMOs appearing in the literature [2, 3] originates from the chitinolytic bacterium *Serratia marcescens*, and in 2010 it was shown that this protein, originally named chitin-binding protein, or CBP21, is an enzyme that cleaves the β-(1 → 4) glycosidic bonds in chitin through oxidation [1]. In addition to the early works showing the importance of proteins now known to be LPMOs in chitin and cellulose depolymerization [3, 4], LPMOs have been found in a wide range of organisms in all domains of life, predominantly in bacteria and fungi but also in viruses [5], oomycetes [6], and invertebrates [7], with activities spanning across a vast array of carbohydrate substrates [6, 8–10]. LPMOs are relatively small copper-dependent redox enzymes that occur as single-domain enzymes or as part of multimodular proteins containing one or multiple carbohydrate-binding modules (CBMs) and, occasionally, additional catalytic domains [11]. Carbohydrate-active enzymes (CAZymes) are classified in the CAZy database according to sequence similarity. The CAZy database contains about 100 CBM families [12], whereas LPMOs are classified in auxiliary activity (AA) families 9–11 and 13–17 [13].

Oxidative cleavage of a glycosidic bond by an LPMO requires reduction of the catalytic mono-copper site, from the inactive [LPMO–Cu (II)] state to the active [LPMO–Cu (I)] state, by an electron donor, typically a small molecule reductant, such as ascorbate or gallic acid. The reduced active state then reacts with an oxygen co-substrate to generate an oxygen species strong enough to abstract a hydrogen atom from the scissile glycosidic bond. Originally, LPMOs were thought to use molecular oxygen (O_2_), but recent data indicate that LPMOs rather act as peroxygenases, using hydrogen peroxide (H_2_O_2_) as their co-substrate [14–19]. It is worth noting that exogenous addition of H_2_O_2_ is not a requirement, due to formation of H_2_O_2_ resulting from the reductant-oxidizing oxidase activity of the LPMO and/or abiotic (i.e., enzyme independent) oxidation of the reductant that takes place in reaction mixtures containing reductant and molecular oxygen [20, 21]. When acting on cellulose, LPMOs incorporate an oxygen atom at the C1 carbon (C1 oxidation) or the C4 carbon (C4 oxidation). This destabilizes the β-(1 → 4)-linked glycosidic bond, leading to its cleavage and formation of an aldonolactone in equilibrium with the aldonic acid or a 4-ketoaldose in equilibrium with the 4-hydroxyaldose, respectively [22, 23]. AA9 LPMOs can exhibit exclusive C1- or C4-oxidation activity, or a combination of both [24].

CBMs are small non-catalytic domains with the primary role of navigating catalytic domains towards the carbohydrate substrate. CBMs are divided into three types based on structural and functional characteristics: type A binds to polysaccharide surfaces, type B binds to single glycan chains, and type C binds to glycan chain ends [12]. Most CBM-containing LPMOs have type A CBMs, for example, a cellulose-binding CBM1, which is commonly found appended at the C-terminus of fungal AA9 LPMOs with known or predicted activity on cellulose. Studies with truncated versions of CBM-containing LPMOs have shown that removal of the CBM leads to weaker substrate binding and decreased cellulose degradation [25–32]. As has been observed for cellulases [33], the role of the CBM is more apparent at lower substrate concentrations [27, 28]. Removal of the CBM does not seem to affect the oxidative regioselectivity of the LPMO [29–31], although this has been reported in one case [28]. Several studies indicate that the linkers connecting the LPMO and CBM domains also affect LPMO functionality [30, 32, 34].

Importantly, in the case of LPMOs, the presence of a CBM also affects enzyme stability because proximity to the substrate increases the chance that available H_2_O_2_ is used productively in a substrate-cleaving peroxygenase reaction, rather than in a non-productive futile peroxidase reaction that may cause oxidative damage to the LPMO [11, 14, 27, 35, 36]. Complicating things further, the level of available H_2_O_2_, and, thus, the rate of an LPMO reaction in absence of exogenously added H_2_O_2_, may also be affected by the presence of a CBM, since substrate binding hampers the oxidase reaction [21], as recently discussed by Stepnov et al. [37].

Typically, the catalytic activity of LPMOs is assessed by examining soluble products generated in reductant-driven enzyme reactions. Assessment of the impact of LPMO action on the fiber fraction is much more challenging and less common. Exploring enzymatically degraded fibers in more depth could shed light on how LPMOs work [38–41], including the impact of CBMs. We have recently developed analytical tools to assess the impact of enzyme treatment on cellulose fibers layer by layer, along the fiber cross section [42]. These tools are based on sequential limited dissolution of cellulose fibers after labeling the carbonyl groups that occur at reducing ends and at C4-oxidized ends emerging after LPMO treatment. Both this study and a preceding comparative study of eleven LPMOs acting on three different cellulose allomorphs [43] have revealed interesting effects of the presence of a CBM, which was especially visible when comparing fiber modification by a C4-oxidizing two-domain fungal LPMO called *Nc*AA9C and its CBM-truncated counterpart (*Nc*AA9C-N). To better understand the impact of the CBM, building on these previous results [42, 43], we have expanded our comparative analysis of the action of full-length and truncated *Nc*AA9C. We assessed the oxidase activity of the *Nc*AA9C variants and their stability under turnover conditions. Furthermore, we have used the recently developed methods for detailed time-course studies of LPMO-induced alterations in the molar mass and degree of oxidation (carbonyl groups) for various layers of Cellulose I fibers. In light of our previous findings [43], we also assessed whether the impact of the CBM varies depending on the type of cellulose fiber (Cell I, Cell II, or amorphous phosphoric acid-swollen cellulose, PASC) and studied if and how the rather weak substrate binding by the catalytic LPMO domain may be enhanced under turnover conditions.

## Methods

### Reagents and substrates

Standard reagents were supplied by Merck Millipore (Burlington, MA, USA) and Sigma-Aldrich (St. Louis, MO, USA), whereas Bacto Yeast Extract and Bacto Peptone were supplied by BD Biosciences (San Jose, CA, USA). Whatman No. 1 filter paper was acquired from GE Healthcare (China) and used to produce three cellulose allomorphs, namely Cell I, Cell II, and PASC, as described earlier [43]. In brief, Cell I substrate was prepared by cold disintegration following a previously published protocol [44], and washed with 1 mM NaHCO_3_ to produce the sodium form as described by Swerin et al. [45]. Cell II was generated from disintegrated Whatman No.1 paper by swelling the fibers in 18% (*w/w*) NaOH, with subsequent washing with MilliQ water. PASC (amorphous cellulose) was generated using orthophosphoric acid as previously described [46]. Dry matter contents were determined using a Sartorius MA37 moisture analyzer (Sartorius Stedim Biotech GmbH, Goettingen, Germany) [43].

### Protein production and purification

The AA9 LPMOs from *Neurospora crassa*, i.e., *Nc*AA9C (UniProt ID, Q7SHI8), and the truncated variant, *Nc*AA9C-N, were produced in *Pichia pastoris* and purified as described earlier [21, 25] using modified purification protocols, as described by Støpamo et al. [43]. Apo forms of *Nc*AA9C and *Nc*AA9C-N were produced by incubating part of the enzyme stock solutions (approx. 150 µM) with 10 mM ethylenediaminetetraacetic acid (EDTA) overnight at 4 °C, with subsequent removal of EDTA by size exclusion chromatography (SEC) using a HiLoad^™^ 16/600 Superdex^™^ 75 PG column (GE Healthcare) using 50 mM Bis–Tris/HCl buffer, pH 6.5, containing 200 mM NaCl as running buffer. Fractions containing the apo enzyme were pooled and then concentrated while replacing the buffer with 50 mM Bis–Tris/HCl buffer, pH 6.5, using centrifugal filters with 3,000 Da MWCO polyethersulfone (PES) membrane (Sartorius Stedim Biotech GmbH, Goettingen, Germany).

Protein concentrations were determined using Bradford’s method (Bio-Rad protein microassay; Bio-Rad Laboratories, Inc; Hercules, CA, USA) with bovine serum albumin as a standard. The absence of endoglucanase background activity was confirmed by carrying out 200 µL overnight reactions with 1 µM LPMO and 1% (*w/v*) Cell I in 50 mM Bis–Tris/HCl, pH 6.5, and without reductants, at 30 °C and 800 rpm. Soluble reaction products were analyzed by high-performance anion exchange chromatography (HPAEC) as described below, and this analysis did not reveal any cellulose degradation.

### LPMO-induced cellulose fiber oxidation

Cellulose fibers, i.e., 50 mg (dry matter content) of Cell I, Cell II, or PASC wet pulp, were treated with *Nc*AA9C or *Nc*AA9C-N in 5 mL reactions under aerobic conditions, as described by Støpamo et al. [43]. In short, the reaction mixtures contained 1% (*w/v*) cellulose fiber, 0.5 µM LPMO, 50 mM Bis–Tris/HCl, pH 6.5, and 1 mM gallic acid. The reactions were initiated by adding gallic acid and incubated at 30 °C for 8 h, 24 h, or 98 h, with vertical shaking at 250 rpm (independent reactions for each timepoint). Control reactions for analysis of non-treated fibers were set up, leaving out both the LPMO and gallic acid or just the LPMO. Gallic acid was chosen as reductant because preliminary results showed that it led to more stable reactions, likely because, in contrast to ascorbate, the abiotic oxidation of gallic acid is not affected by the release of free copper from damaged LPMOs, as discussed by Stepnov et al. [37, 47].

The progress of product formation was monitored in the 98 h reactions by obtaining 50 µL samples after 4, 8, 16, 24, 32, 45, and 73 h. Enzyme activity in these samples was stopped by incubation at 99 °C for 5 min, after which the samples were filtered using a 96 well filter plate with 0.2 µm PES membrane installed on a vacuum manifold (Merck Millipore). Soluble products were analyzed using HPAEC as detailed below. At the reaction endpoint (8, 24, or 98 h), the reactions were incubated at 99 °C for 5 min. After cooling down the sample on ice, the fiber fraction and the supernatant were separated by centrifugation at 5,000 *g* for 20 min at 4 °C. The liquid fraction was removed by careful pipetting and frozen until further analysis by HPAEC. Protein retained in the pellet was removed as described by Støpamo et al. [43], and the fiber samples were stored in 15 mL 75% (*w/v*) EtOH at 4 °C until further analysis. Untreated cellulose reference fibers were directly submerged in 75% (*w/v*) EtOH.

Reactions were set up with *Nc*AA9C-N to determine the reason for the halt in the production of oxidized oligosaccharides by *Nc*AA9C-N after 24 h. Reactions (with 1.2 mL total volume) were set up in duplicates, containing 1% (*w/v*) Cell I, 0.5 µM LPMO, and 1 mM gallic acid in 50 mM Bis–Tris/HCl buffer, pH 6.5. The reaction mixtures were incubated at 30 °C for 72 h with horizontal shaking at 1000 rpm in an Eppendorf Thermomixer C (Eppendorf, Hamburg, Germany). Samples (60 µL) were withdrawn from the reaction mixtures after 8, 16, 24, 36, and 48 h. After 24 h incubation, immediately after sampling, the reactions were supplemented with 19.6 µL of reagents and/or 69 mg of wet fibers as follows: (1) gallic acid alone (to 1 mM final concentration); (2) *Nc*AA9C-N alone (0.5 µM); (3) gallic acid (1 mM) and *Nc*AA9C-N (0.5 µM); (4) Cell I alone [1% (*w/v*)]; (5) Cell I [1% (*w/v*)] and gallic acid (1 mM); (6) Milli-Q water. Product levels were corrected with dilution factors resulting from sampling and addition of liquids. Additional control reactions were set up without gallic acid or LPMO in the reaction. Samples (including the final reaction mixture at 72 h) were boiled for 5 min to stop the reaction and then filtered using a 96 well filter plate with 0.2 µm PES membrane installed on a vacuum manifold (Merck Millipore). Soluble oxidized products were quantified from the filtrate using HPAEC as described below.

### Analysis of soluble oxidized oligosaccharides

Soluble LPMO products were analyzed and quantified by high-performance anion exchange chromatography with pulsed amperometric detection (HPAEC-PAD) using a Dionex ICS 5000 system (Thermo Scientific, Sunnyvale, CA, USA) equipped with a CarboPac PA200 analytical column (3 × 250 mm) and a guard column (3 × 50 mm), using a previously described 26 min gradient [48]. Before analysis, the samples were treated with 1 µM *Tr*Cel7A (produced and purified as described by Ståhlberg et al. [49]), by incubation overnight at 37 °C, to convert the oxidized cello-oligomers to a mixture of Glc4gemGlc and Glc4gem(Glc)_2_. C4-oxidized standards, Glc4gem(Glc)_2_ and Glc4gemGlc, were produced by treating cello-1,4-β-D-pentaose (Megazyme International, Bray, Ireland) with *Nc*AA9C, as previously described [50]. In short, cleavage of the pentamer by *Nc*AA9C produces a mixture of C4-oxidized dimer (along with native trimer) and C4-oxidized trimer (along with native dimer), the amounts of which were determined by quantification of the generated native cello-oligosaccharides in the reaction mixture.

### Fluorescent labeling of cellulose fibers

To analyze the carbonyl group content in untreated or LPMO-treated cellulose fibers, i.e., reducing-end aldehyde groups and 4-keto groups resulting from C4-oxidation, 20–25 mg dry cellulose samples were reacted with the fluorescent label carbazole-9-carboxylic acid [2-(2-aminooxyethoxy)ethoxy]amide (CCOA), following the method described by Röhrling et al. [51]. The labeled cellulose samples were then washed with deionized water to remove unreacted CCOA. Before dissolution in *N, N*-dimethylacetamide (DMAc)/LiCl [9% (*w/v*)], the solvent was exchanged to DMAc to facilitate complete dissolution.

### Dissolution of cellulose

CCOA-labeled cellulose fibers were dissolved using two stepwise sequential dissolution techniques, as outlined in Fig. S1. Sequential limited dissolution with intermittent filtration (Fig. S1A; ‘Approach II’ in Sulaeva et al. [42]), which generates fractions of individual fiber layers, was applied for *Nc*AA9C-treated and reference fibers. In this process, cellulose (ca. 50 mg) is mixed with, and dissolved in, 1 mL DMAc/LiCl [9% (*w/v*)] for a period of time (= a “step”), after which 1 mL of DMAc is added to stop the dissolution, and the dissolved and non-dissolved material are separated by filtration through a 0.45 µm syringe filter. The non-dissolved material is resuspended in 1 mL DMAc/LiCl [9% (*w/v*)] for further dissolution. This procedure is repeated multiple times (five or six times in this study; Fig. S1), with increasingly longer dissolution periods, until total dissolution is achieved [42].

Furthermore, sequential limited dissolution without the separation of already dissolved fiber fractions by filtration (Fig. S1B; ‘Approach I’ in Sulaeva et al. [42]), which generates fractions with the outer layer of the fibers with varying thickness, was also applied for the Cell I fibers after 24 h LPMO treatment. In this process, the LPMO-treated cellulose (ca. 20–25 mg) is mixed with, and dissolved in, 3.5 mL DMAc/LiCl [9% (*w/v*)] for up to 24 h. After different periods of time (= a “step”), a 0.5 mL sample is drawn from the dissolution and mixed with 0.5 mL of pure DMAc. The dissolved material is separated from the non-dissolved material by filtration of the sample through a 0.45 µm syringe filter. Samples taken at a certain timepoint contain all material solubilized up to that timepoint, which means that the later samples comprise both easily dissolving outer layers and more slowly dissolving inner layers of the cellulose.

Total fiber analysis of the Cell I fibers after 24 h LPMO treatment, the results of which have been reported earlier by Støpamo et al. [43], has been performed with single step dissolution [52]. For this, the CCOA-labeled cellulose samples (ca. 15–20 mg) were incubated with 2 mL DMAc/LiCl [9% (*w/v*)] at room temperature for 24 h. This serves as the reference for whole fiber analysis with regard to molar mass and carbonyl groups.

The liquid samples containing solubilized material were analyzed by SEC as described below.

### SEC/MALLS analysis of molecular weight distribution

The labeled and dissolved cellulose solutions were filtered through a 0.45 µm syringe filter and subsequently subjected to size exclusion chromatography (SEC) as described by Sulaeva et al. [52]. In brief, the SEC system included four PLgel MIXED-A columns (20 µm, 7.5 × 300 mm; Agilent, Santa Clara, CA, USA) coupled in series. Detection was performed with a multi-angle laser light scattering (MALLS) detector (λ = 488nm; Wyatt Dawn DSP; Wyatt Technology, CA, USA), a fluorescence detector (excitation at 290 nm, emission at 340 nm; TSP FL2000; Thermo Fisher Scientific, Waltham, MA, USA), and a refractive index (RI) detector (Shodex RI-71; Showa Denko K.K., Isesaki, Gunma, Japan). The utilized eluent was DMAc/LiCl [0.9% (*w/v*)], filtered through a 0.02 μm filter.

Chromeleon 7, Astra 6, and Grams software suites were used to interpret the data. The MALLS and RI data enabled the determination of molecular weight distributions and calculation of molecular weight averages, including number-average (M_n_), weight-average (M_w_), and z-average (M_z_) molar masses and key polymer metrics, such as the dispersity (Đ; defined as the M_w_ to M_n_ ratio) and the degree of polymerization (DP_w_; defined as the M_w_ (in Da) divided by the molecular weight of an anhydroglucose unit). A refractive index increment of 0.140 mL/g was used to quantify the amount of cellulose in the DMAc/LiCl [0.9% (*w/v*)] eluent from the RI peak integral. The carbonyl group content, expressed in µmol/g fiber, was determined based on [51].

### Protein binding to cellulose

An enzyme binding experiment with no reductant has been reported previously [43] and is included in the below for comparative purposes and the sake of completion.

Enzyme binding reactions involving reductants were conducted in two different manners. In one series of experiments, done with the truncated enzyme only, the reaction mixtures contained 2 µM LPMO, 1%, 2%, or 5% (*w/v*) Cell I or Cell II, and 0.1 mM gallic acid or ascorbate in 50 mM Bis–Tris/HCl, pH 6.5. In reactions not containing reductants, an equivalent volume of Milli-Q water was used. As a reference, a reaction with *Nc*AA9C was included without reductant. Reactions were set up in duplicate and were incubated at 30 °C for 45 min in an Eppendorf Thermomixer C (Eppendorf) with 800 rpm horizontal shaking. Samples were filtered as described above. The protein concentration in the filtrates was determined using an optimized variant of the Bradford protein assay, which uses the ratio of absorbances at 590 nm and 450 nm [53]. The amount of non-bound LPMO in the reactions was compared to an appropriately diluted LPMO stock solution corresponding to 100% free enzyme. Furthermore, a standard curve bovine serum albumin was routinely included to verify the linear range of the assay.

In another set of experiments, we used normal turnover conditions, with 1 mM ascorbate as reductant. The reaction mixtures contained 5 µM LPMO and 1% (*w/v*) substrate (Cell I) in 50 mM Bis–Tris/HCl (pH 6.5) and were incubated at 30 °C with horizontal shaking at 1000 rpm. Ascorbate was added at different timepoints (at *t* = 0 min and/or *t* = 120 min), as indicated in the Results and discussion section. The substrate was allowed to swell for 2 h in the reaction solution prior to addition of LPMO and reductant. For each timepoint, a separate reaction was run, and each timepoint was performed in triplicates (so, three independent reactions per timepoint). In this case, unbound protein was quantified by SDS–PAGE analysis of filtered supernatants, followed by quantification of the LPMO band by densitometry with a Gel Doc EZ Imager and Mini PROTEAN TGX Stain-Free SDS–PAGE gels (Bio-Rad, Hercules, CA, USA), as described earlier [54]. In brief, the percentage of free protein was determined from the relative band intensity to a standard solution of known concentration of the respective LPMO. Linearity in the detection range without oversaturation was ensured prior to sample analysis. Samples and standards were filtered using a 96-well 0.2 µm PES filter plate operated with a vacuum manifold (Merck Millipore) prior to analysis.

### Oxidase activity

Formation of H_2_O_2_ by *Nc*AA9C and *Nc*AA9C-N was assessed using a previously described adapted variant [55] of the HRP/Amplex Red assay [21]. The reaction mixtures included 100 µM Amplex Red, 1% (*v/v*) DMSO, 1 mM L-ascorbate, and 5 U/mL lyophilized horseradish peroxidase (HRP) type II (Sigma-Aldrich, St. Louis, MO, USA) in 50 mM Bis–Tris/HCl, pH 6.5. The reactions were set up with freshly copper-saturated LPMOs and apo enzymes by mixing 50 µL of a premix solution containing HRP and Amplex Red in the reaction buffer with 40 µL protein solution in a transparent 96 well microtiter plate and initiated by adding 10 µL of 10 mM ascorbate solution [55]. The final LPMO concentration was 0.5 µM. Control reactions were set up with an ultrafiltrate of the pre-diluted copper-saturated LPMO stock solution (equivalent to 0.5 µM enzyme in the reaction), 0.5 µM CuSO_4_, or Milli-Q water instead of the LPMO. Ultrafiltrates were obtained by using centrifugal filters with 3,000 Da MWCO low-binding PES membrane (VWR, Radnor, PA, USA). Other control reactions were set up without reductant. A standard curve was prepared with H_2_O_2_ (0–20 µM) solutions including 1 mM ascorbate, as described by Stepnov et al. [55]. All reactions were incubated at 30 °C, and apparent H_2_O_2_ formation was determined by monitoring the absorbance at 563 nm every 20 s using a Thermo Scientific^™^ Varioskan^™^ LUX multimode microplate reader (Thermo Scientific). Rates were determined from the linear parts of the progress curves.

## Results and discussion

### Reaction conditions and hydrogen peroxide production

When conducting LPMO reactions aerobically in the presence of reductant, catalysis is limited by the in situ production of H_2_O_2_, which results from both the abiotic oxidation of the reductant and the oxidase activity of the LPMO. In the case of AA9 LPMOs, under the pH and reductant conditions used in this study, the rate of the oxidase reaction may surpass that of abiotic reductant oxidation [55]. However, the contribution of the oxidase reaction diminishes in the presence of substrate [21, 56, 57]. While assessing the impact of the substrate on in situ H_2_O_2_ production is challenging experimentally, it is evident that this impact is more pronounced when the catalytic domain of the LPMO binds tightly to the substrate. The removal of the CBM, as expected and demonstrated further below, reduces substrate binding and may have two effects in LPMO reactions with substrate [37]: it enhances LPMO-catalyzed in situ production of H_2_O_2_, while also potentially increasing damaging peroxidase reactions (i.e., non-productive turnover of H_2_O_2_ by non-substrate-bound LPMOs).

**Fig. 1:**
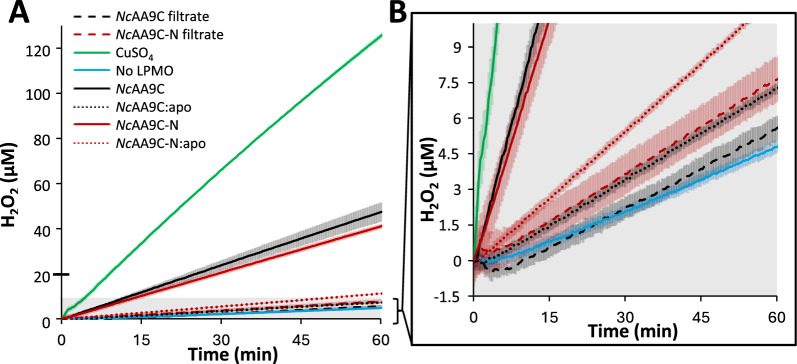
H_2_O_2_ formation by full-length and truncated *Nc*LPMO9C. **B** shows an enhanced version of the area shaded in grey in  **A**. Reaction mixtures contained 1 mM ascorbate, 100 µM Amplex Red, 5 U/mL HRP, and 1% (*v/v*) DMSO in 50 mM Bis–Tris/HCl, pH 6.5, and were supplemented with 0.5 µM copper-saturated or apo-LPMO, 0.5 µM CuSO_4_, the ultrafiltrates of copper-saturated LPMOs corresponding to 0.5 µM enzyme (denoted as “filtrate”), or Milli-Q water (“No LPMO”), as indicated in the figure. The reactions were incubated at 30 °C with continuous monitoring of resorufin formation at 563 nm. Resorufin levels were transferred to H_2_O_2_ levels using a standard curve, as outlined in the Methods section. All reactions in the panels were performed in triplicates; the standard deviations are shown as translucent bars. Control reactions conducted without ascorbate showed no formation of H_2_O_2_

To verify whether the CBM affects H_2_O_2_ formation in the absence of substrate, we quantified H_2_O_2_ production in reactions with ascorbate using the Amplex Red assay [21, 55]. Figure 1 shows that *Nc*AA9C and *Nc*AA9C-N exhibit similar oxidase activities with rates amounting to approximately 1.5 min^−1^. Control reactions using Cu (II) instead of LPMO showed a rate of 4.2 min^−1^ (Fig. 1). These rates are in the same order of magnitude as previously reported rates using similar reaction conditions [55]. Control reactions with (copper-free) apo-enzymes showed that H_2_O_2_ production was strongly reduced; the remaining activity can be attributed to the abiotic oxidation of the reductant. In addition, control reactions with ultrafiltrates of the preparations of copper-saturated enzyme confirmed that these enzyme preparations were devoid of free copper.

### LPMO-catalyzed solubilization and oxidation of Cell I fibers

**Fig. 2:**
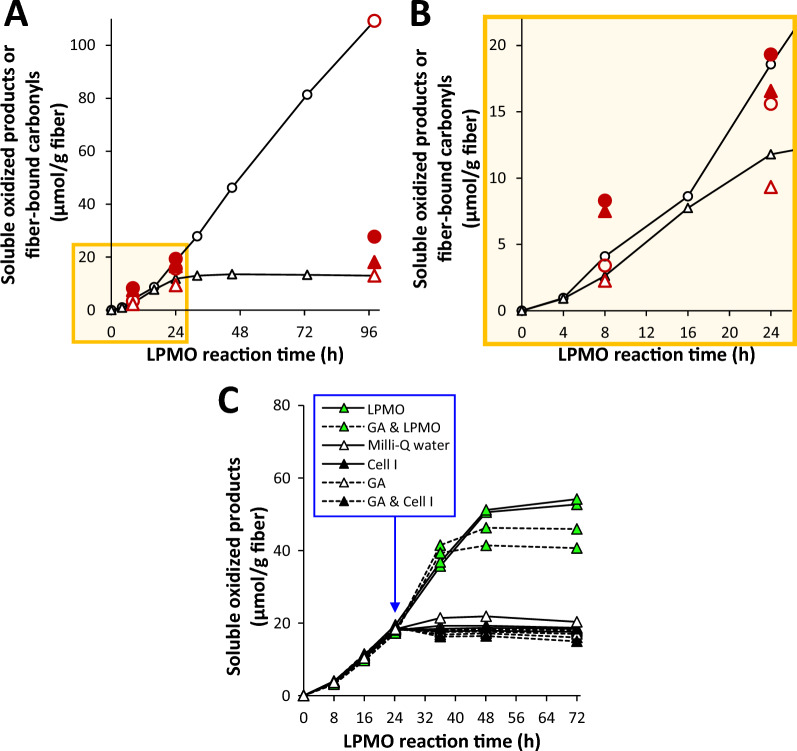
Cellulose solubilization and oxidation by *Nc*AA9C (circles) and *Nc*AA9C-N (triangles). **A** Open symbols show soluble oxidized products determined by HPAEC-PAD, whereas filled symbols show the carbonyl content of the fiber fractions determined by CCOA-SEC/MALLS after complete dissolution of the fibers in a single step. The larger red symbols show end-point data for independent 8 h, 24 h and 98 h reactions. The progress curves depict formation of solubilized oxidized products in the 98-h LPMO reactions. Both soluble oxidized products and fiber carbonyls are expressed in µmol per gram of substrate. For practical reasons, the reactions (total volume, 5 mL), which included fiber analysis, were performed only once per timepoint; note that the fact that the levels of soluble products determined for the 8 h and 24 h reactions (open, colored symbols) align well with the progress curves of the 98 h reactions adds confidence to the data. An alternative dataset, showing the same overall trends, is presented in Fig. S2. **B** Close-up view of the early reaction timepoints in **A**. **C** Product formation in control reactions with *Nc*AA9C-N (same conditions as in **A**) to which various components were added after 24 h in amounts equivalent to those used at the start of the reaction. These reactions (total volume, 1.2 mL) were performed in duplicates, and each reaction is shown by a separate progress curve. The reactions contained 1% (*w/v*) Cell I, 0.5 µM LPMO, and 1 mM gallic acid (GA) in 50 mM Bis–Tris/HCl, pH 6.5, and were incubated at 30 °C and 250 rpm (for reactions in **A**–**B**) or 1000 rpm (for reactions in **C**). Data for **A **and** B** are reproduced from an earlier study [43]

Our earlier study comparing 11 AA9 LPMOs, including *Nc*AA9C and its truncated CBM-free variant [43], revealed differences between the two *Nc*AA9C variants in terms of fiber oxidation. Analysis of the release of soluble products over time in reactions incubated for up to 98 h revealed that the two enzyme variants had similar initial catalytic rates [43], as shown in Fig. 2A, B (black lines). However, while the full-length enzyme showed steady (linear) product generation throughout the 98 h experiment, the production of soluble products ceased after about 24 h in the reaction with *Nc*AA9C-N. An alternative dataset, following the accumulation of soluble LPMO products in reactions conducted at similar (5 mL) scale, shows the same overall trends (see Fig. S2). To determine the reason for the plateauing of product formation by *Nc*AA9C-N, we conducted control experiments by supplying the reaction with substrate, enzyme, and reductant alone or in combinations (Fig. 2C). These experiments showed that the cessation of product release by *Nc*AA9C-N was solely due to enzyme inactivation, since product formation could only be restored when fresh enzyme was added.

Analysis of the fiber fractions after 8, 24, and 98 h of incubation, using complete (single-step) dissolution revealed further trends, as described previously in [43]. As also presented in Fig. 2A, B, up to 24 h, the fiber fractions (filled symbols in Fig. 2A, B) generated by the two enzymes were oxidized to similar extents. In line with the observed inactivation of *Nc*AA9C-N, oxidation of the fiber fraction by *Nc*AA9C-N hardly increased after 24 h. For the still active full-length enzyme, however, the fiber fraction became only slightly more oxidized after 24 h despite the continuous accumulation of soluble oxidized products. The ratio of soluble and insoluble oxidized products (compare open and closed symbols in Fig. 2; see also Fig. S3) increased over the course of reaction for both enzymes as the reaction proceeded, although this trend was more striking for the full-length enzyme. Despite initially being lower, after 98 h, the amount of soluble oxidized products far superseded that of the insoluble oxidized products in the reaction with the full-length enzyme (Figs. 2A, S3). The observation that the amount of oxidized soluble products increased linearly between 24 and 98 h, whereas the increase in the degree of oxidation of the fiber fraction was much more limited, has a logical explanation: if a cellulose chain that is cut once by the LPMO is cut again nearby, leading to the generation of a short oligomeric oxidized product that becomes solubilized, the degree of oxidation of the fiber fraction will not change.

A closer examination of the early phase of the reaction (i.e., up to about 24 h, which marks the end of the linear product formation period by *Nc*AA9C-N and precedes its apparent inactivation; see Figs. 2B, S3) reveals a difference between the two enzyme variants: after 24 h, the ratio of soluble to insoluble products was higher (by 40–45%) for the full-length enzyme (colored circular symbols in Fig. 2B) compared to the truncated enzyme (colored triangular symbols in Fig. 2B). This observation aligns well with the notion that a CBM-containing LPMO, which is to some extent “immobilized” on its substrate, is likely to perform multiple cleavages in a confined region of the fiber surface, which increases the likelihood of the same cellulose chain being cleaved multiple times, leading to the generation of soluble oligomeric products [27]. An independent experiment with other batches of substrate and enzymes showed a similar difference after 23 h of reaction (Figs. S2, S3).

### Localization of enzyme action by the *Nc*AA9C variants

To further investigate differences between how the two LPMO variants modify the cellulose fiber, Cell I fibers treated with *Nc*AA9C or *Nc*AA9C-N (for 8, 24, or 98 h) were solubilized with a recently established sequential dissolution method (Fig. S1A; [42]), which dissolves the fibers layer-by-layer, until completely solubilized. This method allows determining the localization of LPMO action along the fiber cross section. Furthermore, we analyzed the Cell I fibers after 24 h LPMO treatment, together with untreated reference fibers, with an alternative dissolution method (Fig. S1B; [42]), which dissolves the fibers for various durations without separation of the previously dissolved outer fiber fractions. For comparative purposes, data obtained previously with single-step dissolution [43], which dissolves the complete fiber, is included in the discussion. The process involving fluorescence labelling, dissolution, and SEC/MALLS analysis of the fibers is both demanding and time-consuming, and hence was conducted only once for each LPMO reaction timepoint. Consequently, a direct one-to-one comparison of individual data points has limited reliability, and the results should be interpreted by looking at overarching trends. In our discussion, we primarily focus on the 8 h and 24 h LPMO reaction points, since until 24 h, the LPMO reaction had progressed well, while the impact of inactivation of *Nc*AA9C-N was still limited (Fig. 2). When interpreting the results, it is important to consider that shorter polymers may dissolve faster, which means that the separation between fiber layers from ‘surface’ to ‘core’ may vary with the extent of depolymerization resulting from enzyme action. Note that the first dissolution timepoints (0–5 min) of the two sequential dissolution approaches used, representing the fiber surface, are largely comparable as the two dissolution reactions only differ in the volume of DMAc/LiCl [9% (*w/v*)] added to the fiber sample. Similarly, the last dissolution timepoint (0–24 h) of the sequential dissolution method without separation of the previously dissolved outer fiber fractions corresponds to the complete fiber, which can also be obtained using single-step dissolution (0–24 h). The high similarities between these comparable values, obtained with two different dissolution methods, both for Cell I fibers after 24 h LPMO treatment (Table 1) and for the untreated reference fibers (Table S1) add confidence to our analyses.

**Table 1:** Characteristics of the fiber fractions of Cell I fiber after 24 h LPMO treatment obtained using sequential and single-step dissolution methods

LPMO	Dissolution method (fraction)	Dissolution intervals	M_n_ (kDa)	M_w_ (kDa)	M_z_ (kDa)	Đ (M_w_/M_n_)	DP_w_	C = O (µmol/g)
*Nc*AA9C	A (individual layers)	0–5 min	28.2	80.8	236	2.87	498	108
		5–20 min	58.2	139	246	2.38	855	42.5
		20–60 min	104	203	304	1.95	1251	19.5
		1–2 h	137	275	428	2.01	1699	15.0
		2–4 h	198	350	528	1.76	2159	8.73
	A (Fiber core)	4–24 h^a^	238	427	624	1.80	2634	1.08
	B (outer layer of increasing thickness)	0–5 min	26.3	95.3	224	3.62	588	99.9
		0–20 min	28.9	126	349	4.37	779	67.3
		0–60 min	61.6	186	355	3.01	1145	35.7
		0–2 h	78.3	246	457	3.14	1518	23.5
		0–4 h	93.8	271	479	2.89	1671	18.5
	B (complete fiber)	0–24 h^a^	125	327	545	2.62	2016	14.3
	C (complete fiber)	0–24 h^b^	129	314	549	2.43	1937	19.3
*Nc*AA9C-N	A (individual layers)	0–5 min	30.1	82.9	236	2.75	511	88.7
		5–20 min	54.3	129	233	2.37	794	35.7
		20–60 min	93.1	184	276	1.98	1135	19.0
		1–3 h	156	285	420	1.82	1759	7.85
	A (fiber core)	3–24 h^a^	224	386	554	1.72	2378	2.06
	B (outer layer of increasing thickness)	0–5 min	32.4	85.7	228	2.65	200	73.9
		0–20 min	48.6	124	259	2.54	762	54.4
		0–60 min	72.6	190	342	2.61	1171	26.3
		0–2 h	74.2	202	368	2.72	1245	25.5
		0–4 h	82.2	218	398	2.66	1346	23.6
	B (Complete fiber)	0–24 h^a^	112	274	468	2.44	1690	16.4
	C (Complete fiber)	0–24 h^b^	114	265	450	2.33	1633	16.6

**Fig. 3:**
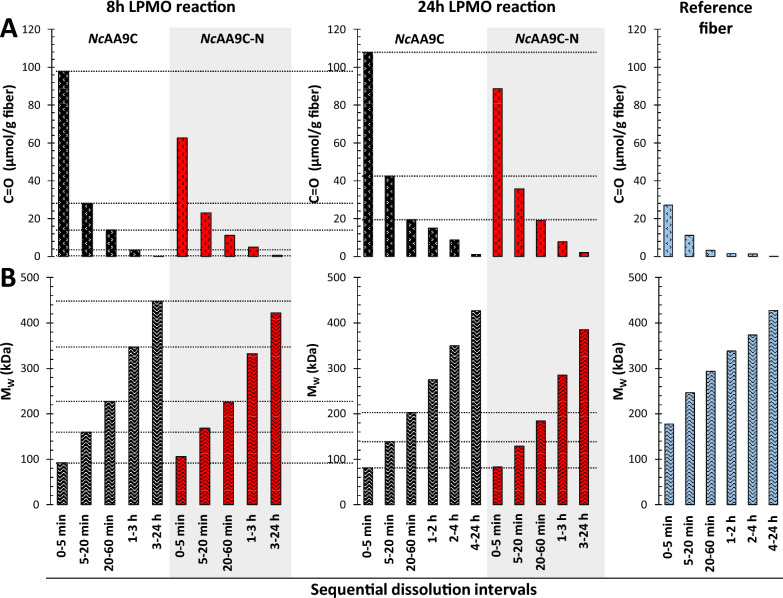
Sequential dissolution analysis of LPMO-treated Cell I. The figure shows the carbonyl content (**A**) and the weight-average molecular weight [M_w_; (**B**)] of the fiber fraction in LPMO reactions run for 8 h (left) or 24 h (middle) and of the untreated reference Cell I fiber (right). The fiber fraction was solubilized in DMAc/LiCl [9% (*w/v*)] solution. At each sampling point, dissolved fibers were removed by filtration and new solvent was added to continue the dissolution. Dissolution times for **A** and **B **are therefore expressed in intervals at the bottom of **B** (note that two slightly different sets of time intervals were used, as explained in Fig. S1). Black bars represent fibers treated with *Nc*AA9C, red bars represent fibers treated with *Nc*AA9C-N, and blue bars represent the untreated Cell I reference fiber. The data for the untreated fiber correspond well to previously established average values [58]. Horizontal dotted lines correspond to values for the reactions with *Nc*AA9C and are meant to facilitate comparisons between identical dissolution times (lines drawn only for fully comparable samples, i.e., samples with exactly the same dissolution time)

Fibers were dissolved using sequential limited dissolution with intermittent filtration (denoted as ‘A’; Fig. S1A) or without separation of fiber fractions by filtration (denoted as ‘B’; Fig. S1B) or using single-step dissolution (denoted as ‘C’), as described in the Methods. The number- (M_n_), weight- (M_w_), and z-average (M_z_) molecular weights, as well as the dispersity (Đ), degree of polymerization (DP_w_), and carbonyl content (C = O), were calculated from SEC/MALLS analyses. “Dissolution intervals” refer to the time periods during which layers of fibers were partially or completely dissolved, resulting in the generation of fiber layers or the complete fiber, respectively. Data for the fiber fractions treated with LPMO for 8 h and 98 h are provided in Table S2. Data for untreated fibers are provided in Table S1. M_w_ and carbonyl values are plotted in Fig. 3

^a^Data previously published in Sulaeva et al., 2024 [42]

^b^Data previously published in Støpamo et al., 2024 [43]

The experiments using sequential dissolution methods (Fig. 3; Tables 1, S2) showed several expected general features. As the LPMO reaction proceeds, the M_w_ of the treated cellulose fibers is reduced while their carbonyl content increases. Comparison of the various dissolution times shows that the early dissolving cellulose chains, likely representing the fiber surface, have a lower M_w_ and a higher carbonyl content than the later-dissolving fractions. The datapoints after 24 h of LPMO reaction show that, overall, the full-length and truncated enzyme variants oxidized the fiber to similar extents. This is apparent from the final 0–24 h dissolution datapoints of the sequential dissolution method without separation of the previously dissolved outer fiber fractions and the data for single-step complete fiber dissolution in Tables 1, S2. Interestingly, despite a similar degree of overall fiber oxidation by the two enzyme variants after 8 h of LPMO treatment (Fig. 2A), the surface (i.e., early dissolving) fraction of the LPMO-treated fibers contained 56% more carbonyl groups for the full-length enzyme compared to the CBM-free variant (Fig. 3; Table S2).

While the carbonyl content of the early dissolving fiber fractions is clearly higher for reactions with the full-length LPMO, this trend diminishes, and may even seem to become reversed, for the later dissolving fractions, suggesting that the truncated enzyme can penetrate deeper into the fiber. Such type of difference between the truncated and full-length LPMO may also be derived from the M_w_ values in the 8 h reaction data (Fig. 3B; Table S2), which show that, while M_w_ of the early dissolving fibers is slightly higher for the truncated enzyme compared to the full-length enzyme, the truncated enzyme gives a larger reduction in M_w_ for late-dissolving fibers that are closer to the core. This latter phenomenon, i.e., more reduction in M_w_ of the deeper layers when using the truncated enzyme, is also visible for the 24 h reaction data (Fig. 3B).

Another difference between the two enzyme forms becomes apparent when comparing the number-average (M_n_, Table 1) and weight-average (M_w_, Table 1) molecular weights of the early dissolving fiber layers. For the earliest dissolving (0–5 min) fraction of the 24 h-treated fibers, the reductions in M_n_ and M_w_ were similar for the two LPMOs, while for the later dissolving fractions (5–20 and 20–60 min) these reductions are clearly higher when using the truncated LPMO. The same trend has also been reported earlier for M_n_ and M_w_ of the complete (0–24 h in Table 1) fiber fractions [42, 43]. In cases where a large polymer chain is cleaved randomly (and far away from the chain ends), which in the present case would generate non-soluble, “longer” products, M_w_ will be decreased. Thus, our data are compatible with the notion that the CBM-containing enzyme generates a higher fraction of short products (resulting in higher M_w_), while cleavages by the truncated enzyme are more evenly spread over the fiber (resulting in lower M_w_).

### The impact of the CBM on substrate preferences

**Fig. 4:**
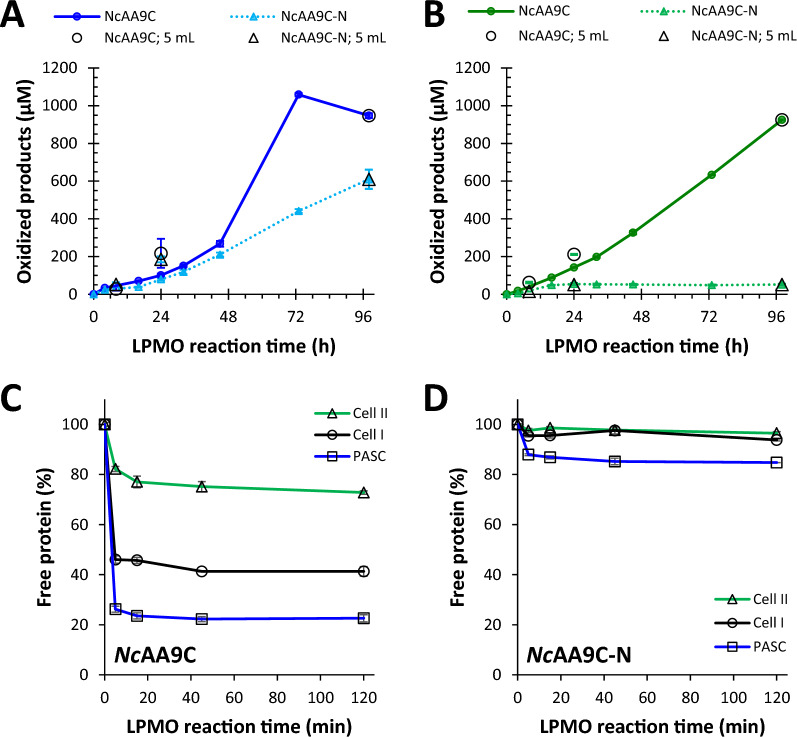
Substrate-dependent impact of the CBM on binding and catalytic activity of *Nc*AA9C. **A** and **B **show the formation of soluble oxidized products in reactions of *Nc*AA9C (circles, solid line) or *Nc*AA9C-N (triangles, dotted line) with PASC (**A**) and Cell II (**B**). The larger, open symbols show oxidized products detected in the filtrates of independent 5 mL reactions run for 8, 24, or 98 h; the filled symbols show the accumulation of solubilized oxidized products in the 98-h LPMO reactions. Reactions contained 1% (*w/v*) substrate, 0.5 µM LPMO, and 1 mM gallic acid in 50 mM Bis–Tris/HCl buffer, pH 6.5, and were incubated at 30 °C with 250 rpm horizontal shaking. In control reactions lacking the reductant, no oxidized products were detected (not shown). Reactions were run in duplicates, and error bars represent standard deviation. The data corresponding to product formation for Cell I by the *Nc*AA9C variants are shown in Fig. 2A. **C** and **D** show binding of the LPMOs to Cell I (circles, black line), Cell II (triangles, green line), and PASC (squares, blue lines) in 200 µL reactions using the same reaction conditions as in **A, B**, but without addition of reductant and with 2 µM LPMO, and horizontal shaking at 800 rpm. Protein binding is expressed as the fraction (percentage) of soluble protein in the filtrate of the reaction solutions. Reactions were run in triplicates, and error bars represent standard deviation. This figure is based on data that have been published previously [43]

In a recent comparative study of eleven LPMOs [43], we have shown that LPMOs, including *Nc*AA9C and *Nc*AA9C-N, differ in terms of their ability to bind to and degrade the different cellulose allomorphs. As to *Nc*AA9C, both the full-length and the truncated enzyme were able to oxidize and solubilize all three substrates (data shown in Fig. 4A), with C4-specific oxidative regioselectivity. Still, the progress curves showed large, substrate-dependent variation. The CBM-containing full-length enzyme maintained activity for most or all of the 98 h reaction period and generated approximately the same amount of products with all three substrates, indicating that the reaction was limited by access to the co-substrate, H_2_O_2_, and not by substrate affinity, the effective substrate concentration, and/or enzyme inactivation. Binding studies showed that the full-length enzyme binds to all three cellulose substrates, albeit with different efficiencies (Fig. 4B**)**, where the latter, notably, may reflect a difference in the effective substrate concentration rather than a true difference in affinity. In any case, the binding data show that the impact of the CBM on substrate binding clearly depends on the cellulose-type.

In contrast, and in line with the data shown for Cell I in Fig. 2, the truncated protein was rapidly inactivated in the reactions with Cell I and, notably more so, Cell II (Fig. 4B). This suggests that the truncated enzyme is limited by its inability to productively bind to Cell I and Cell II, which, as outlined above, will lead to rapid inactivation resulting from off-pathway redox reactions. Indeed, binding assays showed minimal binding of the truncated enzyme to these two cellulose forms (Fig. 4D). On the other hand, when using PASC, the truncated LPMO showed binding (Fig. 4D) and generated soluble oxidized products for most of the 98 h reaction period (Fig. 4A). It is interesting to note that both LPMO forms bind to (amorphous) PASC, especially in light of the general idea that the main function of LPMOs is to attack hard-to-degrade crystalline substrates. *Nc*AA9C is special in that it shows high activity on soluble cello-oligomers, i.e., single glycan chains [59]), which may explain why the enzyme works so well on PASC.

Of note, the shapes of the progress curves for the full-length enzyme (Figs. 2, 4A, B) differ, with PASC standing out in that product formation ceases around 73 h, indicative of enzyme inactivation. C4-oxidized products are unstable and will start deteriorating when the LPMO is no longer consuming available H_2_O_2_ [60], explaining the decrease in product levels between 73 and 98 h. Clearly, the type of cellulose has an impact on LPMO function, and this impact is modulated by the CBM.

Of note, the truncated *Nc*AA9C-N enzyme is capable of degrading Cell I and Cell II, while showing weak binding to this substrate. Importantly, the binding studies displayed in [43] and Fig. 4 were done in the absence of reductant, i.e., with the LPMO in the Cu (II) state. It has been shown previously, for full-length *Nc*AA9C, that reduction of the enzyme improves binding to cellulose [61]. In fact, Kracher et al. [61] concluded that reduction may even “initiate” binding, which may indeed be true for productive binding of the catalytic domain to the substrate, but certainly not for CBM-mediated binding, as shown in Fig. 4. Binding studies involving a reductant are complicated because substrate degradation will occur, with concomitant changes in the fiber structure and potential enzyme binding sites. Enzyme inactivation under turnover conditions is another complicating factor.

**Fig. 5:**
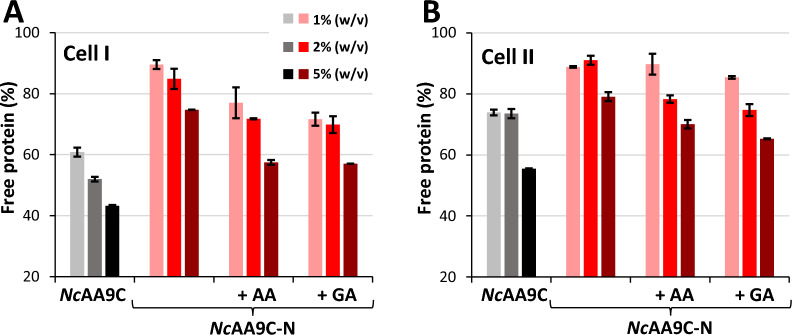
Substrate-dependent binding of the LPMO and the impact of reduction. The figure displays the fraction of free protein in solution after a 45 min incubation of 2 µM LPMO with 1%, 2%, or 5% (*w/v*) Cell I (**A**) or Cell II (**B**) in 50 mM Bis–Tris/HCl buffer, pH 6.5. Data for the full-length enzyme, *Nc*AA9C, is shown with black and grey bars, while data for the truncated form, *Nc*AA9C-N, is shown with red and pink bars. The binding reactions with the truncated enzyme included reactions with 0.1 mM ascorbate (AA) or 0.1 mM gallic acid (GA). Error bars reflect the standard deviations from two independent experiments. All reactions were incubated at 30 °C with horizontal shaking at 800 rpm in 200 µL reaction volumes

To further explore the impact of reduction and better represent turnover conditions, we studied binding of the truncated *Nc*AA9C-N to Cell I and Cell II fibers, in the presence of ascorbate or gallic acid. The experiments were conducted using a tenfold lower reductant concentration compared to previous experiments, in an attempt to slow down the enzymatic reaction. We also included experiments with higher substrate concentrations, 2% and 5% (*w/v*), compared to the standard amount of 1% (*w/v*) that was used in the experiments described above. Binding experiments with the full-length enzyme were included as a control and confirmed that the CBM-harboring enzyme binds better to Cell I compared to Cell II (Fig. 5). The Cu (II) form of the truncated enzyme, which did not show significant binding to 1% (*w/v*) Cell I and Cell II (Fig. 4), showed binding to these substrates at the higher substrate concentrations, and, importantly, binding was enhanced upon reduction with either ascorbate or gallic acid (Fig. 5). At 1% (*w/v*) and in the presence of reductant, the truncated enzyme binds clearly better to Cell I (Fig. 5), which aligns well with the better catalytic performance of this enzyme with 1% (*w/v*) Cell I, compared to 1% (*w/v*) Cell II (Fig. 4).

**Fig. 6:**
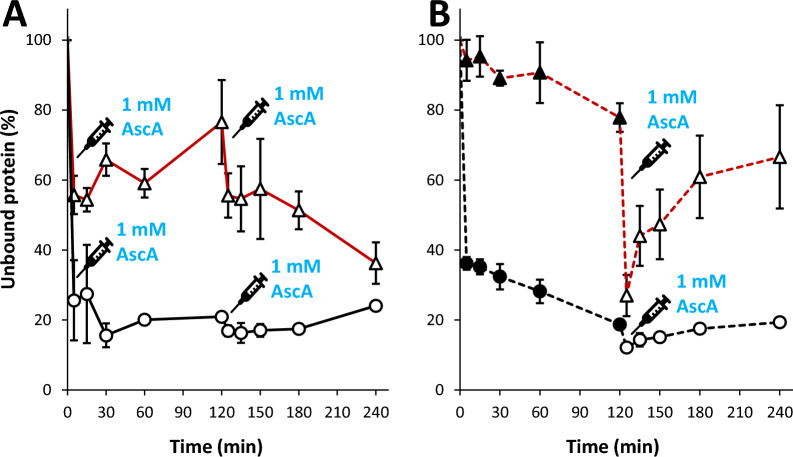
Progress curves of LPMO binding and the effect of 1 mM ascorbate. The figure shows binding by *Nc*AA9C (circles, black color) and *Nc*AA9C-N (triangles, red color) to Cell I. Ascorbate (AscA) was added at *t* = 0 and/or *t* = 120 min, as indicated in the figure. **A** Initial reduction of the LPMOs, followed by a subsequent addition of ascorbate after 120 min. **B** Binding by the non-reduced LPMOs, followed by addition of ascorbate after 120 min. Reaction conditions: 5 µM LPMO and 1% (*w/v*) substrate in 50 mM Bis–tris/HCl (pH 6.5), incubated at 30 °C, 1000 rpm. Each timepoint corresponds to a separate reaction, and each timepoint was performed in triplicates

To gain further insight into substrate binding and the impact of the reductant, we conducted binding experiments with both enzyme variants under turnover conditions, i.e., in the presence of 1 mM reductant. Since these experiments are demanding and since Fig. 5 did not show significant differences between the effects of gallic acid and ascorate, we only studied effects of the latter reductant. These are complicated experiments since the enzymes are very active and may at the same time suffer from inactivation, especially in case of the truncated variant. Indeed, the results (Fig. 6) showed large standard deviations, and some binding progress curves were oddly shaped. Still, the data show very clear trends: the CBM-containing enzyme binds strongly to the substrate, regardless of the addition of reductant, while binding of the CBM-free enzyme is weak but is strongly enhanced in the presence of reductant. Of note, despite high standard deviations, the immediate effect of adding reductant on binding of the truncated enzyme is clear (e.g., in Fig. 6B). This effect is much faster than the expected rate of enzyme inactivation [35], thus excluding the possibility that the apparent effects of the reductant on binding are due to oxidative (irreversible) destruction of the enzyme. The fact that drastic irreversible enzyme destruction is not happening in this time scale is also demonstrated by the slow release of protein in the period subsequent to addition of ascorbate.

### Concluding remarks

In this work, we show that the presence of the CBM1 in *Nc*AA9C drastically improves binding towards all tested forms of cellulose, amorphous (PASC), natural (Cell I), and alkali-swollen (Cell II) cellulose. We also show that CBM-mediated binding differs between these substrates, and that the CBM is essential for significant binding of the non-reduced LPMO to Cell I and Cell II. It is important to separate between “general”, and potentially even non-productive, binding by the CBM and productive binding of the catalytic domain that leads to catalysis. In accord with and expanding previous observations [61], we show that binding of the catalytic domain of *Nc*AA9C to crystalline cellulose forms is promoted by reduction of the copper site. Nevertheless, this effect of reduction alone is not sufficient: while both enzyme forms were able to oxidize and solubilize the three cellulose types, the truncated variant performed much worse for the two crystalline celluloses, due to rapid enzyme inactivation. Increased susceptibility to autocatalytic inactivation is a well-known effect of removing CBMs from LPMOs [11, 14, 28, 36].

The sequential dissolution data provide a glimpse of how LPMO action affects cellulose fibers from surface to core, and of the impact of the CBM. Taken together, the single-step and sequential dissolution revealed that the two enzyme variants oxidize the fiber to similar extents (at 24 h), while the truncated enzyme leads to greater reduction in the M_w_ of the fiber relative to the full-length enzyme. This highlights differences in the mode of oxidation by the two enzyme forms and shows that cuts by the CBM-free enzyme are more evenly spread through the fiber compared to the CBM-containing full-length enzyme, in line with conclusions drawn from other studies using different methods [27, 62]. Interestingly, the sequential dissolution data indicate that the truncated enzyme can penetrate deeper into the fiber, thus introducing relatively more oxidation and cleavage in the fiber core. In conclusion, our study demonstrates the capability of LPMOs to modify cellulose fibers from surface to core and reveals how changes in enzyme modularity can yield varied materials. While the implications of these findings for LPMO-based cellulose fiber engineering remain to be explored, it is clear that the presence of a CBM plays an important role.

### Supplementary Information

Supplementary Material 1.

## Data Availability

Data are provided within the manuscript or supplementary information files.

## Abbreviations

AA: Auxiliary activity
CBM: Carbohydrate-binding module
CCOA: Carbazole-9-carboxylic acid [2-(2-aminooxyethoxy)ethoxy] amide
DMAc: *N*, *N*-Dimethylacetamide
DMSO: Dimethyl sulfoxide
DP: Degree of polymerization
EDTA: Ethylenediaminetetraacetic acid
HPAEC: High-performance anion exchange chromatography
HRP: Horseradish peroxidase
LPMO: Lytic polysaccharide monooxygenase
MALLS: Multi-angle laser light scattering
MWCO: Molecular weight cutoff
PAD: Pulsed amperometric detection
PASC: Phosphoric acid-swollen cellulose
PES: Polyethersulfone
RI: Refractive index
SEC: Size exclusion chromatography
