# Supplementary material for: The impact of the carbohydrate-binding module on how a lytic polysaccharide monooxygenase modifies cellulose fibers
Source: Biotechnol Biofuels Bioprod. 2024 Aug 24;17:118. doi: 10.1186/s13068-024-02564-8 (PMC11344300; doi:10.1186/s13068-024-02564-8)
Supplement: Supplementary file 1 — Supplementary Material 1. [file 13068_2024_2564_MOESM1_ESM.pdf]

Supporting information

Title: The impact of the carbohydrate-binding module on how a lytic polysaccharide monooxygenase modifies cellulose fibers

Authors: Fredrik G. Støpamo[1], Irina Sulaeva[2], David Budischowsky[2], Jenni Rahikainen[3], Kaisa Marjamaa[3], Kristiina Kruus[3,4], Antje Potthast[2], Vincent G.H. Eijsink[1], Anikó Várnai[1,*]

Affiliations:

1) Norwegian University of Life Sciences (NMBU), Ås, Norway
2) University of Natural Resources and Life Sciences (BOKU), Vienna, Austria
3) VTT Technical Research Centre of Finland, Espoo, Finland
4) Aalto University, Espoo, Finland

E-mail addresses:

Fredrik G. Støpamo	fredrik.gjerstad.stopamo@nmbu.no
Irina Sulaeva	irina.sulaeva@boku.ac.at
David Budischowsky	david.budischowsky@boku.ac.at
Jenni Rahikainen	jenni.rahikainen@vtt.fi
Kaisa Marjamaa	kaisa.marjamaa@vtt.fi
Antje Potthast	antje.potthast@boku.ac.at
Kristiina Kruus	kristiina.kruus@aalto.fi
Vincent G.H. Eijsink	vincent.eijsink@nmbu.no
Anikó Várnai	aniko.varnai@nmbu.no

[*]**Corresponding author.** E-mail: aniko.varnai@nmbu.no; Telephone: +47-6723-2569.

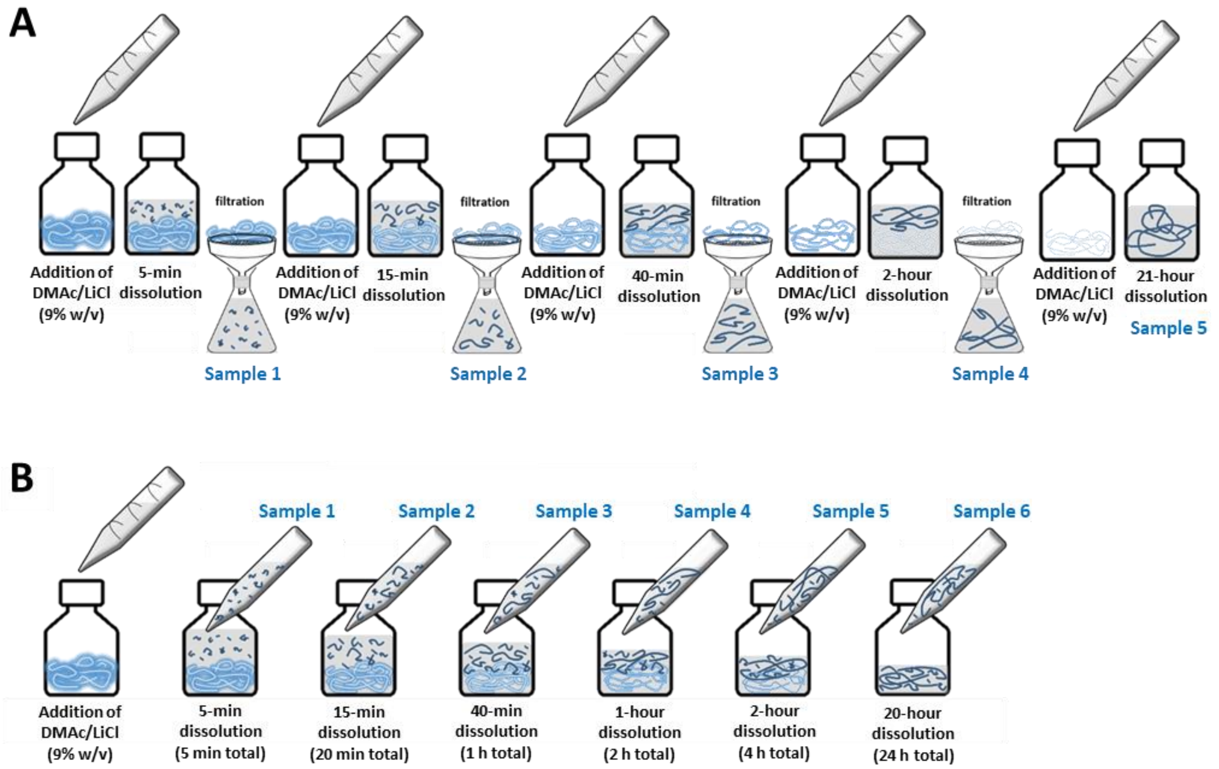

Figure S1. Schematic overview of the stepwise cellulose dissolution methods in DMAc/LiCl (9% (*w/v*)). In panel **A**, permeate and retentate fractions are separated through intermittent filtration, with the retentate being subjected to further solubilization in fresh DMAc/LiCl (9% (*w/v*)). Soluble fractions from each cycle were analyzed using SEC/MALLS. The method is described in detail (denoted as 'Approach II') by Sulaeva et al. [42]. Note that two different protocols were used in this study with slightly different time ranges, either as displayed in the Figure, giving the time intervals 0–5 min, 5–20 min, 20–60 min, 1–3 h, and 3–24 h, or with one extra step (5 min, 15 min, 40 min, 1 h, 2 h, 20 h), giving the time intervals 0–5 min, 5–20 min, 20–60 min, 1–2 h, 2–4 h and 4–24 h. In panel **B**, fiber dissolution is carried out continuously for a total of 24 h dissolution time, with intermittent sampling of the dissolution reaction. These samples are diluted and filtered before SEC/MALLS analysis, as described in the Methods section. The method is described in detail (denoted as 'Approach I') by Sulaeva et al. [42].

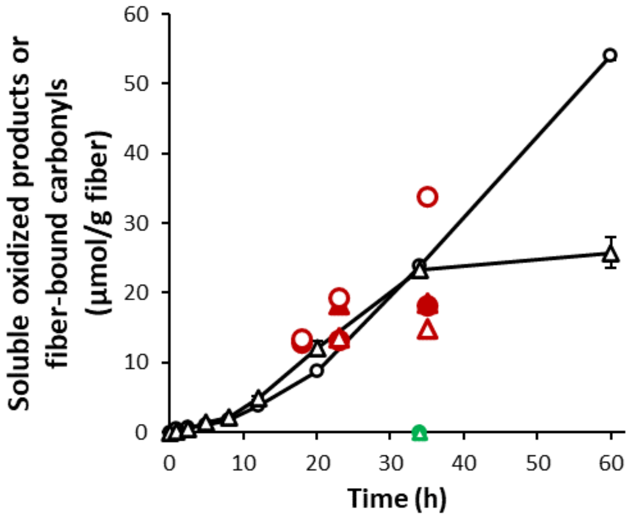

Figure S2. Cellulose solubilization and oxidation by *Nc*AA9C (circles) and *Nc*AA9C-N (triangles). Open symbols show soluble oxidized products determined by HPAEC-PAD, whereas filled symbols show the carbonyl content of the fiber fractions determined by CCOA-SEC/MALLS after complete dissolution of the fibers in a single step. Progress curves for *Nc*AA9C (circles) and 9C-N (triangles) in small scale reaction setups (black symbols and lines, in triplicates per time point, with each time point in a separate reaction tube), or large scale 5 mL reactions (red symbols). Reactions with enzymes but no GA, or without enzymes but with GA, yielded no soluble oxidation products (in triplicates for each reaction at 34 h; green symbols). Reaction conditions: 0.5 µM LPMO, 1 mM GA, 1% (*w/v*) Cell I fiber, 50 mM Bis-Tris/HCl, pH 6.5, 30 °C, shaking at 800 rpm (250 rpm for the large scale reactions). This dataset was generated using other substrate and enzyme batches than those used to generate **Fig. 2** in the main manuscript. The observed trends are essentially the same as those visible in **Fig. 2** of the main manuscript.

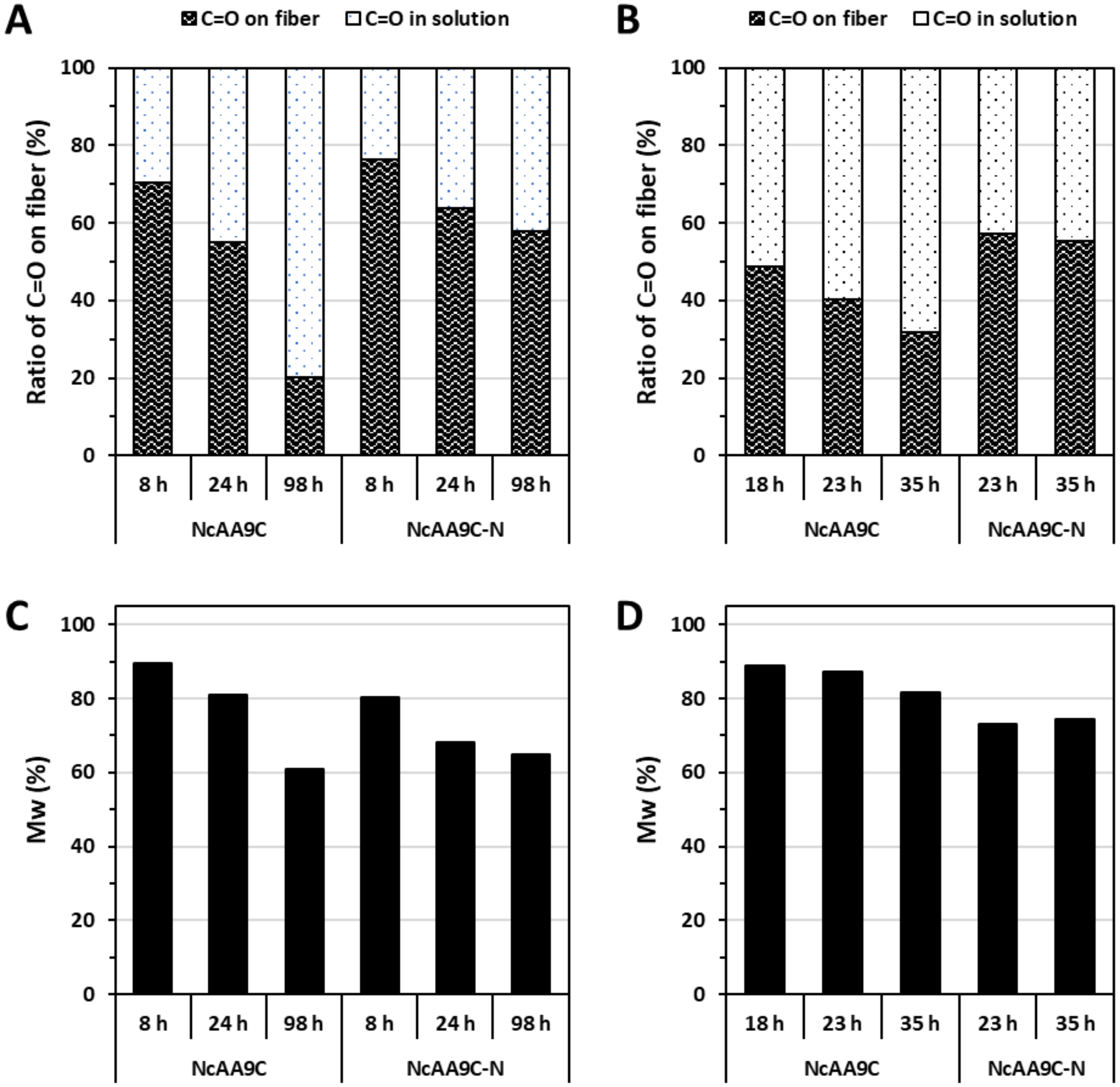

Figure S3. Changes in the ratio of carbonyl groups located in the fiber fraction vs in solution and in the average molecular mass of cellulose during *Nc*AA9C treatment of Cell I fibers. Panels **A** and **B** show the distribution of carbonyl groups generated during LPMO reaction between the fiber and soluble fractions in the large scale reactions shown in **Figs. 2** and **S2**, respectively. Panels **C** and **D** show the corresponding decrease in M_w, obtained with single-step dissolution, in the same samples. This figure highlights that the two enzyme forms differ in terms of the ratio of soluble and insoluble (i.e., in the fiber) oxidized products, while the relative amount oxidized groups that are solubilized in the reaction increases over time for both *Nc*AA9C variants. Oxidized products were quantified as the total amount of soluble C4-oxidized products and as the carbonyl content of the fiber. To form the ratios of LPMO-generated carbonyl groups in the fiber and soluble fractions, the amount of carbonyl groups present in the control fiber was subtracted from the amount of carbonyl groups present in the LPMO-treated fiber samples. Panels **A** and **C** were calculated from data presented in Støpamo et al. [43].

Table S1. Characteristics of fiber fractions of untreated Cell I fiber obtained after sequential and single-step dissolution methods. Fibers were dissolved using sequential limited dissolution with intermittent filtration (denoted as 'A'; **Fig. S1A**) or without separation of fiber fractions by filtration (denoted as 'B'; **Fig. S1B**) or using single-step dissolution (denoted as 'C'), as described in the Methods. The number average molecular weight (M_n), the weight average molecular weight (M_w) and the z-average molecular weight (M_z) were derived from SEC/MALLS analyses. The dispersity, Đ, equals M_w/M_n. The carbonyl content was derived from the SEC/MALLS analysis as described in the Methods. The DP_w was calculated by dividing the M_w by the molecular weight of an anhydroglucose unit. "Dissolution intervals" refer to the time periods during which layers of fibers were partially or completely dissolved, resulting in the generation of fiber layers or the complete fiber, respectively.

**Fiber**	**Dissolution method (fraction)**	**Dissolution intervals**	**M_n (kDa)**	**M_w (kDa)**	**M_z (kDa)**	**Đ (M_w/M_n)**	**DP_w**	**C=O (µmol/g)**
Cell I	A (Individual layers)	**0–5 min**	**50.8**	**178**	**357**	**3.49**	**1095**	**27.2**
		5–20 min	80.0	246	426	3.08	1520	11.1
		20–60 min	123	294	485	2.39	1811	3.27
		1–2 h	160	338	551	2.12	2087	1.52
		2–4 h	175	374	581	2.14	2304	1.36
	A (Fiber core)	**4–24 h[a]**	**239**	**428**	**663**	**1.79**	**2639**	**0.07**
	B (Outer layer of increasing thickness)	**0–5 min**	**51.7**	**181**	**363**	**3.50**	**1116**	**19.3**
		0–20 min	77.9	235	438	3.02	1449	16.9
		0–60 min	121	286	475	2.37	1766	5.71
		0–2 h	135	315	543	2.34	1945	3.90
		0–3 h	140	321	544	2.30	1979	2.97
		0–4 h	157	335	547	2.14	2064	1.93
	B (Complete fiber)	**0–24 h[a]**	**181**	**355**	**573**	**1.96**	**2191**	**0.21**
	C (Complete fiber)	**0–24 h[b]**	**214**	**389**	**592**	**1.82**	**2401**	**0.13**

[a] Data previously published in Sulaeva et al., 2024 [42].

[b] Data previously published in Støpamo et al., 2024 [43].

Table S2. Characteristics of fiber fractions obtained after 8 h (top) and 98 h (bottom) LPMO reactions using sequential dissolution with intermittent filtration. The number average molecular weight (M_n), the weight average molecular weight (M_w) and the z-average molecular weight (M_z) were derived from SEC/MALLS analyses. The dispersity, Đ, equals M_w/M_n. The carbonyl content was derived from the SEC/MALLS analysis as described in the Methods. The DP_w was calculated by dividing the M_w by the molecular weight of an anhydroglucose unit. "Dissolution intervals" refer to the time periods during which fibers were partially dissolved, resulting in the generation of fiber layers (see **Fig. S1A**). Averages were calculated for the 0–60 min ("surface") or the 1–24 h ("core") dissolution intervals. Note that the truncated enzyme is largely inactive after 24 h. Data for the 24 h reaction is provided in the main manuscript (**Table 1**).

**8 h reaction** LPMO	**Dissolution intervals**	**M_n (kDa)**	**M_w (kDa)**	**M_z (kDa)**	**Đ (M_w/M_n)**	**DP_w**	**C=O (µmol/g)**
LPMO							
***Nc*AA9C**	0–5 min	33.3	92.5	243	2.78	570	97.9
	5–20 min	79.7	160	259	2.00	984	28.1
	20–60 min	115	227	339	1.97	1401	14.0
	1–3 h	173	347	513	2.01	2143	3.30
	3–24 h	226	448	654	1.98	2762	0.07
Average	*Surface*	*76.1*	*160*	*280*	*2.25*	*985*	*46.7*
Average	*Core*	*199*	*398*	*583*	*2.00*	*2452*	*1.69*
***Nc*AA9C-N**	0–5 min	39.4	106	254	2.68	652	62.7
	5–20 min	79.4	169	271	2.12	1040	23.1
	20–60 min	125	226	322	1.80	1393	11.2
	1–3 h	173	332	497	1.93	2049	4.96
	3–24 h	262	423	605	1.61	2606	0.62
Average	*Surface*	*81.4*	*167*	*282*	*2.20*	*1028*	*32.3*
Average	*Core*	*217*	*377*	*551*	*1.77*	*2328*	*2.79*

**98 h reaction** LPMO	**Dissolution intervals**	**M_n (kDa)**	**M_w (kDa)**	**M_z (kDa)**	**Đ (M_w/M_n)**	**DP_w**	**C=O (µmol/g)**
LPMO							
***Nc*AA9C**	0–5 min	27.5	63.9	178	2.33	394	142
	5–20 min	42.0	97.3	199	2.32	600	58.8
	20–60 min	79.2	147	221	1.85	905	28.8
	1–2 h	133	236	371	1.78	1453	20.8
	2–4 h	140	315	508	2.24	1941	15.6
	4–24 h	163	380	581	2.34	2344	3.44
Average	*Surface*	*49.6*	*103*	*199*	*2.17*	*633*	*76.5*
Average	*Core*	*145*	*310*	*487*	*2.12*	*1913*	*13.3*
***Nc*AA9C-N**	0–5 min	28.5	73.6	212	2.58	454	95.0
	5–20 min	46.7	108	220	2.32	668	52.6
	20–60 min	76.4	149	227	1.95	917	23.3
	1–3 h	131	248	368	1.90	1531	9.81
	3–24 h	202	368	542	1.82	2269	2.42
Average	Surface	*50.5*	*110*	*220*	*2.28*	*680*	*56.9*
Average	Core	*167*	*308*	*455*	*1.86*	*1900*	*6.12*
